# Oxidative Stress-Induced Diseases via the ASK1 Signaling Pathway

**DOI:** 10.1155/2012/439587

**Published:** 2012-05-13

**Authors:** Mayumi Soga, Atsushi Matsuzawa, Hidenori Ichijo

**Affiliations:** Laboratory of Cell Signaling, Graduate School of Pharmaceutical Sciences, The University of Tokyo, 7-3-1 Hongo, Bunkyo-ku, Tokyo 113-0033, Japan

## Abstract

Apoptosis signal-regulating kinase 1 (ASK1) is a mitogen-activated protein kinase (MAPK) kinase kinase that activates the downstream MAPKs, c-Jun N-terminal kinase (JNK) and p38. ASK1 is activated by various types of stress, such as oxidative stress, endoplasmic reticulum stress, and infection, and regulates various cellular functions. Recently, it has been reported that ASK1 is associated with various diseases induced by oxidative stress. In this review, we introduce recent findings of the regulatory mechanisms of ASK1 and the oxidative stress-induced diseases mediated by the ASK1 signaling pathway.

## 1. Introduction

 Cells are exposed to various types of external and internal stresses and need to respond to these stresses to maintain homeostasis. The mitogen-activated protein kinase (MAPK) pathway is one of the intracellular signaling systems that regulate various cellular functions, such as proliferation, differentiation, and apoptosis. Each MAPK pathway consists of three classes of protein kinases: MAPK kinase kinase (MAP3K), MAPK kinase (MAP2K), and MAPK. MAP3K phosphorylates and thereby activates MAP2K, and activated MAP2K, in turn, phosphorylates and activates MAPK. Among MAPKs, c-Jun N-terminal kinase (JNK) and p38 MAPK respond to various types of stress, including reactive oxygen species (ROS), osmotic pressure, tumor necrosis factor-*α* (TNF-*α*), and endoplasmic reticulum (ER) stress, and regulate apoptosis, inflammation, and morphogenesis through the phosphorylation of various target molecules [1]. MAP3Ks control the activation status of MAPKs, and thus, MAP3Ks are important for the regulation of various cellular responses.

 Apoptosis signal-regulating kinase 1 (ASK1) is a member of the MAP3K family, which activates the MAPK kinase 4 (MKK4)/MKK7-JNK and MKK3/6-p38 pathways [1, 2]. ASK1 is preferentially activated in response to various types of stress, such as ROS, TNF-*α*, lipopolysaccharide (LPS), and ER stress, and has pivotal roles in a wide variety of cellular responses, including apoptosis, differentiation, and inflammation [2–7]. Therefore, the excessive activation and dysregulation of ASK1 are closely linked to various diseases. Here, we focus on the molecular mechanisms of ASK1 activation and the involvement of ASK1 in oxidative stress-induced diseases.

## 2. Mechanisms of ROS-Induced ASK1Activation

 ASK1 forms a high molecular mass complex termed the ASK1 signalosome [8]. Within the signalosome, ASK1 is homooligomerized through its C-terminal coiled-coil (CCC) domain, a process that is critical for ASK1 activation. Thioredoxin (Trx), a redox-responsive protein, is included in the ASK1 signalosome, and the reduced form of Trx binds to the N-terminal region of ASK1 and inhibits its kinase activity. However, the oxidized form of Trx dissociates from ASK1 in response to ROS, and ASK1 is then activated by the autophosphorylation of Thr845 in its kinase domain [3, 9]. Upon ROS-dependent dissociation of Trx from ASK1, ASK1 appears to be tightly oligomerized through its N-terminal coiled-coil (NCC) domain, leading to the full activation of ASK1.

 The ROS-stimulated ASK1 signalosome forms a much higher molecular mass complex than when not stimulated and contains various regulatory factors of ASK1, including TNF-*α* receptor-associated factor 2 (TRAF2), TRAF6, protein phosphatase 5 (PP5), and USP9X [8, 10, 11]. In response to ROS, the adaptor proteins TRAF2 and TRAF6 are recruited to the ASK1 signalosome and positively regulate ASK1 activity by promoting the homophilic interaction of its NCC domain [12]. TRAF2 and TRAF6 promote ASK1-dependent cell death and inflammatory cytokine production downstream of the TNF-*α* receptor and Toll-like receptor 4 (TLR4; a receptor for LPS), respectively. PP5 dephosphorylates the activating phosphorylation site of ASK1 in a ROS-dependent manner and negatively regulates ASK1 activity [10].

**Figure 1:**
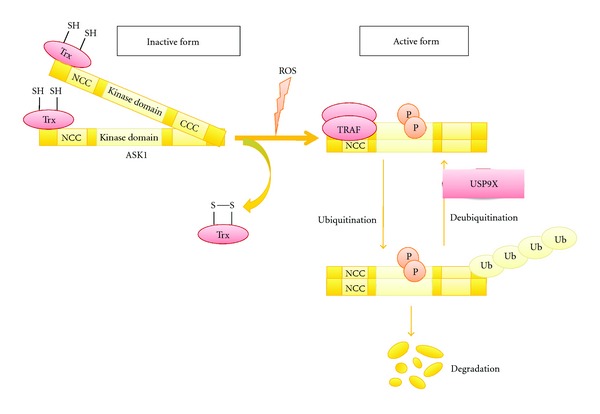
Mechanisms of ROS-induced ASK1 activation. Thioredoxin (Trx), a negative regulator of ASK1, is dissociated from the N-terminal region of ASK1 in response to ROS. Subsequently, TRAF2 and TRAF6 are recruited, thereby fully activating ASK1. The ROS-induced activation of ASK1 results in its ubiquitination and proteasome-dependent degradation. USP9X negatively regulates ASK1 degradation by deubiquitination, leading to the sustained activation of ASK1. CCC: C-terminal coiled-coil domain; NCC: N-terminal coiled-coil domain.

 The USP9X deubiquitination enzyme also binds to ASK1 in response to oxidative stress. The oxidative stress-induced activation of ASK1 results in the ubiquitination and proteasome-dependent degradation of ASK1. Thus, USP9X positively regulates ASK1 activity and ASK1-dependent cell death through the deubiquitination and stabilization of ASK1 [11]. Therefore, ASK1 activity is regulated by both phosphorylation and ubiquitination in response to oxidative stress (Figure 1).

## 3. Cancer and ASK1

 Recently, it has been reported that ASK1 has an important role in skin tumorigenesis [22]. The ROS-induced activation of the ASK1-p38 pathway leads to the production of inflammatory cytokines, such as TNF-*α*, IL-6, and IL-1*β*, in dendritic cells and macrophages [6], and the ASK1-dependent production of inflammatory cytokines was found to be critical for chemically induced skin tumorigenesis during the promotion stage. In this case, ASK1 acts as a promoter of skin tumorigenesis. When ASK1 acts with ASK2, a functional binding partner of ASK1, during the initiation stage of skin tumorigenesis, ASK1 contributes to the induction of ROS-dependent apoptosis in epidermal keratinocytes. Because the ASK1-ASK2 complex functions as a tumor suppressor, the expression level of ASK2 appears to regulate the roles of ASK1 as a tumor promoter and suppressor. It has been observed that the expression of ASK2 was strongly reduced in various human gastrointestinal cancer cells and tissues compared with their normal counterparts [22]. Actually, ASK2 deficiency promotes chemically induced mouse skin tumorigenesis, through the reduction of apoptosis in DNA-damaged epidermal keratinocytes during the initiation stage. ASK2 has been shown to activate ASK1 by direct phosphorylation.

 ASK1 also acts as a tumor suppressor in hepatocarcinogenesis [17]. ASK1 is involved in death receptor-mediated apoptosis through the JNK-mediated phosphorylation of BimEL, a proapoptotic Bcl-2 family member, and the DNA damage-induced upregulation of p21 through the p38 pathway. ASK1 also contributes to the development of gastric cancer [21]; the expression level of ASK1 increased in human gastric cancer, and ASK1-deficient mice had both fewer and smaller tumors than wild-type mice. ASK1 upregulates the expression level of cyclin D1 through AP-1 activation, leading to cell proliferation. Moreover, cyclin D1 elevates ASK1 expression via the Rb-E2F pathway such that this positive feedback loop facilitates the development of gastric cancer. Thus, ASK1 functions as a tumor promoter and also as a tumor suppressor, depending on the cell type and cellular context, through the induction of various cellular responses, such as apoptosis, inflammation, and cell proliferation. Most recently, it has also been reported that frequent somatic mutations in ASK1 in metastatic melanoma were identified by exome sequencing [23]. Several mutations affect the kinase activity of ASK1. The Ile780Phe substitution in the kinase domain of ASK1 almost completely abolishes kinase activity, whereas the Glu663Lys substitution adjacent to the kinase domain reduces weakly, yet significantly, the kinase activity.

## 4. Neurodegenerative Diseases and ASK1

 Alzheimer's disease (AD) is a neurodegenerative disorder characterized by two pathological findings: amyloid-*β* (A*β*) accumulation and neurofibrillary tangles. A*β* is the major component of senile plaques and induces neuronal cell death, and it has been reported that A*β* impairs mitochondrial redox activity and increases the generation of ROS, leading to apoptotic neuronal death. A*β* also activates ASK1 through the generation of ROS and induces JNK-mediated neuronal cell death [13]. It has been observed that A*β*-induced neuronal cell death decreases in ASK1-deficient mice, indicating that ROS-induced ASK1 activation by A*β* is an important step in the pathogenesis of AD.

 Recently, it has also been reported that ASK1-mediated dopaminergic (DA) neuronal cell death is important for Parkinson's disease [14]. The peroxiredoxin 2 (Prx2) antioxidant enzyme inhibits DA toxin 6-hydroxydopamine-(6-OHDA-) induced ASK1 activation by modulating the redox status of Trx and inhibiting the dissociation of Trx from ASK1. Prx2 confers remarkable protection against 6-OHDA-induced DA neuronal loss via the suppression of the ASK1-dependent activation of JNK and p38. In addition, the oxidative stress-mediated activation of the ASK1-JNK pathway is associated with brain ischemia in the hippocampus [15]. Thus, ROS-induced ASK1 activation contributes to the pathogenesis of neurodegenerative diseases.

## 5. Inflammation and ASK1

 ASK1 also responds to biological stresses, such as bacterial and viral infection, and induces inflammation. Bacterial components, such as LPS, are recognized by TLRs and activate the downstream MAPK pathways. ASK1-deficient mice have been shown to be resistant to LPS-induced sepsis shock [6]. The LPS-induced p38 activation and production of inflammatory cytokines were reduced in splenocytes and dendritic cells derived from ASK1-deficient mice. Because LPS-induced p38 activation and cytokine production were suppressed by antioxidants, this implies that LPS-induced ASK1 activation is mediated by ROS generation. These results indicate that ASK1 is important for mammalian innate immunity. In addition, recent research has shown that the activation of the ASK1-p38 pathway through TLRs in glial cells is important for chemokine production in astrocytes and facilitates inflammation and neurotoxicity in multiple sclerosis (MS) [16]. An ASK1 deficiency or ASK1 inhibitor attenuated the sensitivity of experimental autoimmune encephalomyelitis, an animal model of MS, suggesting that ASK1 is a potential therapeutic target for the treatment of MS.

## 6. Cardiac Diseases and ASK1

ASK1 is closely linked to cardiac diseases, such as cardiac hypertrophy, remodeling, and cardiac injury. It has been reported that in the left ventricle, ASK1 is activated by angiotensin-II-(Ang II-) induced ROS generation through the Ang II type 1 (AT1) receptor, resulting in cardiac hypertrophy and remodeling [18, 19]. Ang II-induced cardiac hypertrophy and remodeling, including cardiac hypertrophy-related mRNA upregulation, cardiomyocyte apoptosis, and interstitial fibrosis, were significantly attenuated in ASK1-deficient mice. ASK1 is also involved in Ang II-induced cardiac injury, such as capillary endothelial apoptosis, and a decrease in myocardial capillary density [20]. Cardiac injury was prevented by an AT1 receptor blocker through the inhibition of ROS generation and ASK1 activation in a mouse model of hypertensive decompensated cardiac hypertrophy and heart failure. ASK1 contributes to Ang II-induced cardiac diseases mediated by ROS generation.

## 7. Diabetes and ASK1

 Several studies have revealed that ASK1 is associated with the pathogenesis of diabetes. ASK1 has been shown to negatively regulate insulin receptor substrate-1 (IRS-1), a key mediator in insulin signaling, through JNK-mediated IRS-1 phosphorylation [24]. ASK1 is activated by TNF-*α*-stimulated ROS generation and is an important factor that causes insulin resistance. Hyperglycemia increases oxidative stress in various tissues, and it has been reported that the high glucose-induced activation of ASK1 contributes to endothelial cell senescence, leading to diabetes-related vascular aging mediated by oxidative stress [25]. These results suggest that ROS-mediated ASK1 activation is involved in the pathogenesis of diabetes through the modulation of insulin signaling or cellular senescence.

## 8. Conclusions

 Redox balance is important for the control of cellular responses, and the mode of cell reaction depends on the level of oxidative stress. As described above, the ASK1 signaling pathway is closely linked to various human diseases caused by oxidative stress and redox imbalance through the regulation of various cellular responses, such as apoptosis, inflammation, proliferation, and senescence. Recent studies have shown that ASK1 is a therapeutic candidate for these diseases (summarized in Table 1). ASK1 functions as an initial sensor of ROS generation and plays a pivotal role in signal transduction for the maintenance of homeostasis against redox imbalance. The excessive activation and dysregulation of ASK1 result in a wide range of diseases. Further studies will reveal the pathophysiological roles of ASK1 in oxidative stress-induced diseases, leading to the development of therapeutic strategies.

## Figures and Tables

**Table 1:** ASK1-related diseases and pathologies.

Organ/tissue	Related diseases	Related pathologies	References
Nervous system	Alzheimer's disease	Neuronal death	[13]
	Parkinson's disease	Neuronal death	[14]
	Brain ischemia	Neuronal death	[15]
	Multiple sclerosis	Inflammation	[16]

Liver	Hepatocarcinogenesis	Apoptosis	[17]

Heart	Hypertrophy, remodeling Cardiac injury	Cardiomyocyte apoptosis, interstitial fibrosis Capillary endothelial apoptosis	[18, 19]
			[20]

Stomach	Gastric cancer	Cell proliferation	[21]

Skin	Skin tumorigenesis	Apoptosis, inflammation	[22]

Immune system	Infection	Septic shock	[6]

## Abbreviations

ASK1: Apoptosis signal-regulating kinase 1
MAPK: Mitogen-activated protein kinase
JNK: c-Jun N-terminal kinase
TNF-*α*: Tumor necrosis factor-*α*
ROS: Reactive oxygen species
LPS: Lipopolysaccharide
Trx: Thioredoxin
TRAF: TNF-*α* receptor-associated factor
TLR: Toll-like receptor
Prx: Peroxiredoxin
Ang II: Angiotensin II.
